# Identifying the link between serum VEGF and KL-6 concentrations: a correlation analysis for idiopathic pulmonary fibrosis interstitial lung disease progression

**DOI:** 10.3389/fmed.2023.1282757

**Published:** 2023-12-01

**Authors:** Bin Zhong, Shuixiang Luo

**Affiliations:** Department of Respiratory Medicine, The First Affiliated Hospital of Gannan Medical University, Ganzhou, Jiangxi, China

**Keywords:** IPF-ILD, serum markers, KL-6, VEGF, PaO2

## Abstract

**Background:**

Idiopathic pulmonary fibrosis interstitial lung disease (IPF-ILD) is a progressive lung disease characterized by excessive collagen deposition and fibrotic changes in the lungs. Identifying reliable serum markers that correlate with disease progression is crucial for diagnosis and prognosis.

**Objective:**

This study aimed to explore the association between serum markers KL-6 and VEGF and IPF-ILD. Specifically, it assessed their correlation with PaO2, a measure of pulmonary gas function, to provide diagnostic and prognostic indicators.

**Methods:**

Patients with IPF-ILD were included, and their serum levels of KL-6 and VEGF were measured. Correlations with fibrotic damage and PaO2 were analyzed using statistical methods.

**Results:**

The analysis confirmed a positive correlation between the serum marker KL-6 and the degree of fibrotic damage in IPF-ILD. On the other hand, the serum marker VEGF was found to promote disease progression. In terms of correlation with PaO2, both KL-6 and VEGF demonstrated high sensitivity and specificity. Specifically, the correlation between KL-6 and PaO2 suggests that it can be used as a reliable indicator to assess the status of pulmonary oxygenation function in patients with ILD. The correlation between VEGF and PaO2 helps to understand its role in the progression of IPF-ILD and provides an important basis for predicting patient prognosis.

**Conclusion:**

This study confirmed the correlation between KL-6 and VEGF with IPF-ILD and their association with PaO2. KL-6 and VEGF demonstrated high specificity and sensitivity in diagnosing, monitoring, and predicting prognosis in IPF-ILD. These findings contribute to our understanding of the disease and have clinical implications for diagnosis and prognostication.

## 1 Introduction

Idiopathic pulmonary fibrosis (IPF) is a specific type of interstitial lung disease (ILD) characterized by progressive scarring and fibrosis of the lung tissue (1). The pathogenesis of IPF is complex and incompletely understood, and involves a combination of genetic susceptibility, environmental factors, and an aberrant immune response (2). The prognosis for IPF is poor, and it is still a challenging disease to diagnose despite advances in diagnostics and treatment (3, 4).

For idiopathic pulmonary fibrosis interstitial lung disease (IPF-ILD), recent studies have revealed potential mechanisms and biomarkers associated with disease progression (5–10). Among them, vascular endothelial growth factor (VEGF), a key angiogenic factor, promotes neovascularization, increases vascular permeability, and regulates the expression of cytokines associated with fibrosis, thus driving the progression of IPF-ILD (11). Krebs von den Lungen-6 (KL-6) is mainly expressed in type II alveolar epithelial cells and bronchial epithelial cells. It was found that regeneration of type II epithelial cells was accompanied by a significant increase in KL-6 in the alveolar basement membranes of patients with IPF-ILD, and this increase was positively correlated with the extent of tissue fiber damage (12–15). Although the importance of VEGF and KL-6 in the pathogenesis of ILD has been widely recognized (16), further exploration and validation of their clinical applications in the diagnosis, assessment of severity, and prediction of prognosis in IPF-ILD are needed. Therefore, the aim of this study was to analyze the relationship between VEGF and KL-6 and their associations with related clinical parameters by detecting their expression levels in the serum of IPF-ILD patients in order to explore their roles in IPF-ILD disease progression and the value of their clinical use.

To achieve this goal, we collected clinical data of a group of IPF-ILD patients and their serum samples from real cases in a hospital in China, and applied reliable experimental methods for the determination of VEGF and KL-6. Relevant clinical parameters, such as disease duration and lung function indices, were also collected. By comprehensively analyzing these data, we hope to reveal the potential role of VEGF and KL-6 in the development and prognosis of IPF-ILD, and to provide new ideas and methods for the early diagnosis and treatment of IPF-ILD. In terms of related studies, several studies have explored the changes in the expression of VEGF and KL-6 in IPF-ILD and their relationship with disease progression. However, comprehensive studies on the pathogenesis and clinical application of the two in IPF-ILD have been relatively insufficient, such as lack of respondent operating characteristic (ROC) curve analysis, or longitudinal information regarding course over time, etc. Therefore, this study further deepens the understanding of the roles of these two factors in IPF-ILD and explores their potential applications in the clinic and confirms the important role of serum VEGF and KL-6 in the development of IPF-ILD. Through correlation analysis with clinical parameters such as HRCT, lung function, and blood gas analysis, we conclude that the expression levels of serum VEGF and KL-6 can be used as important indicators for assessing the severity of IPF-ILD, monitoring disease progression and predicting prognosis. These results are expected to provide clinicians with a more accurate diagnosis and the basis for individualized treatment strategies, thereby improving the prognosis and quality of life of ILD patients. It should be noted that due to the limitations of the study sample and data, the results of this study still need further validation and expansion of the sample size to enhance the reliability. In addition, further in-depth mechanistic studies also help to recognize the exact mechanism of serum VEGF and KL-6 in the progress of IPF-ILD.

## 2 Methods

### 2.1 Introduction of the research content

Interstitial pneumonia causes proliferation of type alveolar epithelial cells, which leads to an increase in KL-6 concentration. Moreover, lungs’ basement membrane injury enhanced vascular permeability, allowing KL-6 to reach the circulation. Assessment of pulmonary gas function in patients with IPF-ILD is an important aspect of disease progression and clinical management, and partial pressure of arterial oxygen (PaO2) is one of the measures of the lung’s capacity for gas exchange and oxygenation. In patients with IPF-ILD, lung fibrosis and structural disturbances lead to abnormalities in alveolar ventilation and pulmonary vascular distribution, which in turn affect oxygenation. Therefore, PaO2 is often used as an indicator to assess the status of pulmonary gas function and disease severity in patients.

This study intends to investigate the correlation between serum VEGF, KL-6 and their expression levels, and to further find out the correlation between their expression levels and clinical indexes such as HRCT and lung function blood gas analysis, so as to increase the understanding and diagnostic accuracy of IPF-ILD disease. First, we have collected serum samples from a group of patients with ILD and measure the expression levels of VEGF and KL-6. Next, we performed HRCT scans on these patients to assess the extent of lung lesions and pulmonary function blood gas analysis to assess pulmonary gas exchange.

Subsequently, statistical analyses were used to explore the correlation between the expression levels of serum VEGF and KL-6 with HRCT and pulmonary function blood gas analysis. By analyzing the data, we hope to discover associations between these two biomarkers and clinical indicators, thus providing more insight into the diagnosis and treatment of IPF-ILD.

### 2.2 Subject selection and clinical data collection

Patients with IPF-ILD diagnosed by clinical and imaging examinations were selected as study subjects and screened from the hospital’s database of IPF-ILD patients. Meanwhile, a certain number of healthy individuals were selected as the control group. The basic information of each patient was collected, including age, gender, and disease duration. Peripheral blood samples were collected from each patient and centrifuged to obtain serum samples.

At the beginning of the study, a survey was conducted to determine that a total of 120 patients were treated at the First Affiliated Hospital of Gannan Medical University. Among these patients, 100 patients were diagnosed as idiopathic pulmonary fibrosis-interstitial lung disease (IPF-ILD) patients. To ensure that these samples were randomized and representative, we randomly selected 40 IPF-ILD patients as the study group. In addition, we selected 20 patients with non-ILD disease as a control group. Although we did not purposely control for age-matching or other matching factors of the patients, factors such as patient age were taken into account when performing data analysis to ensure appropriateness in terms of statistical correction. This step was taken to minimize selection bias and to better reflect the actual patient population.

### 2.3 Diagnosis and exclusion criteria

Idiopathic pulmonary fibrosis interstitial lung disease is diagnosed based on a combination of clinical, imaging, pulmonary function, and histopathologic tests, and by an experienced chief physician.

1. Clinical presentations: Patients can experience shortness of breath at rest or after activity. A dry cough or the posterior aspect of the chest may present with a popping sound (Velcro rales), which is the sound produced when the mucous membranes between the lobes of the lungs separate from each other during respiration.
2. Imaging: High-resolution computed tomography (HRCT) of the chest is commonly used to evaluate lung lesions, and IPF-ILD is usually characterized by interstitial fibrotic plaques and honeycombed lung parenchyma.
3. Pulmonary function tests: Pulmonary function tests may reveal restrictive ventilation or pulmonary ventilation dysfunction, including reduced lung volumes, reduced forced expiratory volumes, reduced forced expiratory one-second volumes, and reduced lung diffusion function.
4. Histopathology: Lung tissue samples are obtained by bronchofiberscopy or lung biopsy, etc. After histopathological examination, features of IPF-ILD such as fibrosis and alveolar wall thickening can be observed (17). In addition, confirming the diagnosis of IPF-ILD requires the exclusion of other possible causes of lung pathology, such as occupational exposures, environmental factors, drug-induced lung injury, and cardiac insufficiency. For other diseases, such as chronic obstructive pulmonary disease (COPD), pneumonia, tuberculosis, silicosis, bronchial asthma, bronchiectasis, and acute bronchitis, diagnosis and exclusion need to be made in accordance with the relevant guidelines or criteria to avoid misdiagnosis of these diseases as IPF-ILD (18). Finally, patients need to be ruled out as having a comorbid tumor to ensure a correct diagnosis of IPF-ILD.

### 2.4 Determination of serum VEGF, KL-6 levels

Fasting venous blood of 3 mL was collected from all enrolled patients on the day of consultation or in the early morning of the next day, and the blood specimens were left to stand at room temperature for 1 h and centrifuged at 3,500 *r/min* for 15 *min*, and then the supernatant was separated and extracted, and stored at −80°C for measurement.

Enzyme-linked Immunosorbent Assay (ELISA) was used to determine the levels of VEGF and KL-6 in serum. According to the instructions of the kit, first, the sample serum and standards were added into the corresponding wells of the enzyme plate, and then the enzyme-labeled antibody solution was added, incubated for a certain time and then washed. Next, the substrate solution is added so that it binds to the enzyme and reacts to form a color. Finally, a termination solution is added to terminate the reaction and the absorbance value is read using an enzyme marker. By comparing with the standard curve, the concentrations of VEGF and KL-6 in serum can be calculated. Each sample was duplicated to ensure the accuracy and reproducibility of the assay results.

### 2.5 Statistical methods

The statistical analysis was performed using SPSS 20.0 software. Measurement data were presented as mean ± standard deviation (±s). For continuous variables that followed a normal or slightly skewed distribution with low chi-squaredness, independent samples *t*-tests were used for group comparisons. Nonparametric rank tests were employed for variables that did not meet the criteria for parametric analysis. Pearson linear correlation analysis was conducted to examine the correlations between two groups of continuous variables, while Spearman rank correlation analysis was used for ranked variables. A value of *p* of less than 0.05 was considered statistically significant indicating a significant difference or correlation.

The collected data were analyzed using statistical software. First, the expression levels of VEGF and KL-6 were analyzed with descriptive statistics, including mean, standard deviation, and percentile. Then, the differences in VEGF and KL-6 between the ILD patient group and the control group were compared using appropriate statistical methods (e.g., *t*-test, ANOVA, or nonparametric tests). Correlation analysis is used to assess the relationship between VEGF and KL-6, as well as correlations with clinical parameters (e.g., disease duration, lung function indices). In all statistical analyses, a *p* value of less than 0.05 can be considered statistically significant.

## 3 Results and analysis

The collected data were analyzed using statistical software. Appropriate statistical methods, such as *t*-test, ANOVA, or nonparametric tests, were used to compare the differences in VEGF and KL-6 between groups. In addition, correlation analysis was used to assess the relationship between VEGF and KL-6 and clinical parameters. Further, ROC curve analysis was applied to assess the accuracy of VEGF and KL-6 in the diagnosis of IPF-ILD.

### 3.1 Analysis of experimental data

In this study, we investigated the role and clinical application value of VEGF and KL-6 in the development of IPF-ILD by detecting their expression levels in the serum of IPF-ILD patients. We collected clinical data and serum samples from a group of IPF-ILD patients and utilized reliable experimental methods for the determination of VEGF and KL-6. We also collected relevant clinical parameters, such as disease duration and pulmonary function index PaO2.

First, the mean levels of serum VEGF and KL-6 were compared between the IPF-ILD group and the control group. Using *t*-tests and non-parametric tests, it was determined whether there were significant differences. As shown in Table 1, the results showed that the serum VEGF level in the IPF-ILD group was 117.22 ± 18.68 (*p* < 0.05), which was significantly higher than that in the control group, which was 81.20 ± 7.57 (*p* < 0.05). Similarly, the serum KL-6 level in the IPF-ILD group was 495.07 ± 48.89 (*p* < 0.05), which was significantly higher than the KL-6 level of 300.59 ± 32.34 (*p* < 0.05) in the control group.

**Table 1:** Statistical results of data from the experimental and control groups of IPF-ILD.

	VEGF (*pg/mL*)	KL-6 (*U/mL*)	PaO2 (*mmHg*)	Age
IPF-ILD	117.22 ± 18.68 (*p* < 0.05)	495.07 ± 48.89 (*p* < 0.05)	86.44 ± 5.34 (*p* < 0.05)	54.00 ± 11.00 (*p* < 0.05)
Non-ILD	81.20 ± 7.57 (*p* < 0.05)	300.59 ± 32.34 (*p* < 0.05)	95.93 ± 5.69 (*p* < 0.05)	54.00 ± 16.00 (*p* < 0.05)
Total	105.79 ± 23.04 (*p* < 0.05)	433.23 ± 105.28 (*p* < 0.05)	89.36 ± 7.09 (*p* < 0.05)	54.00 ± 15.00 (*p* < 0.05)

**Figure 1:**
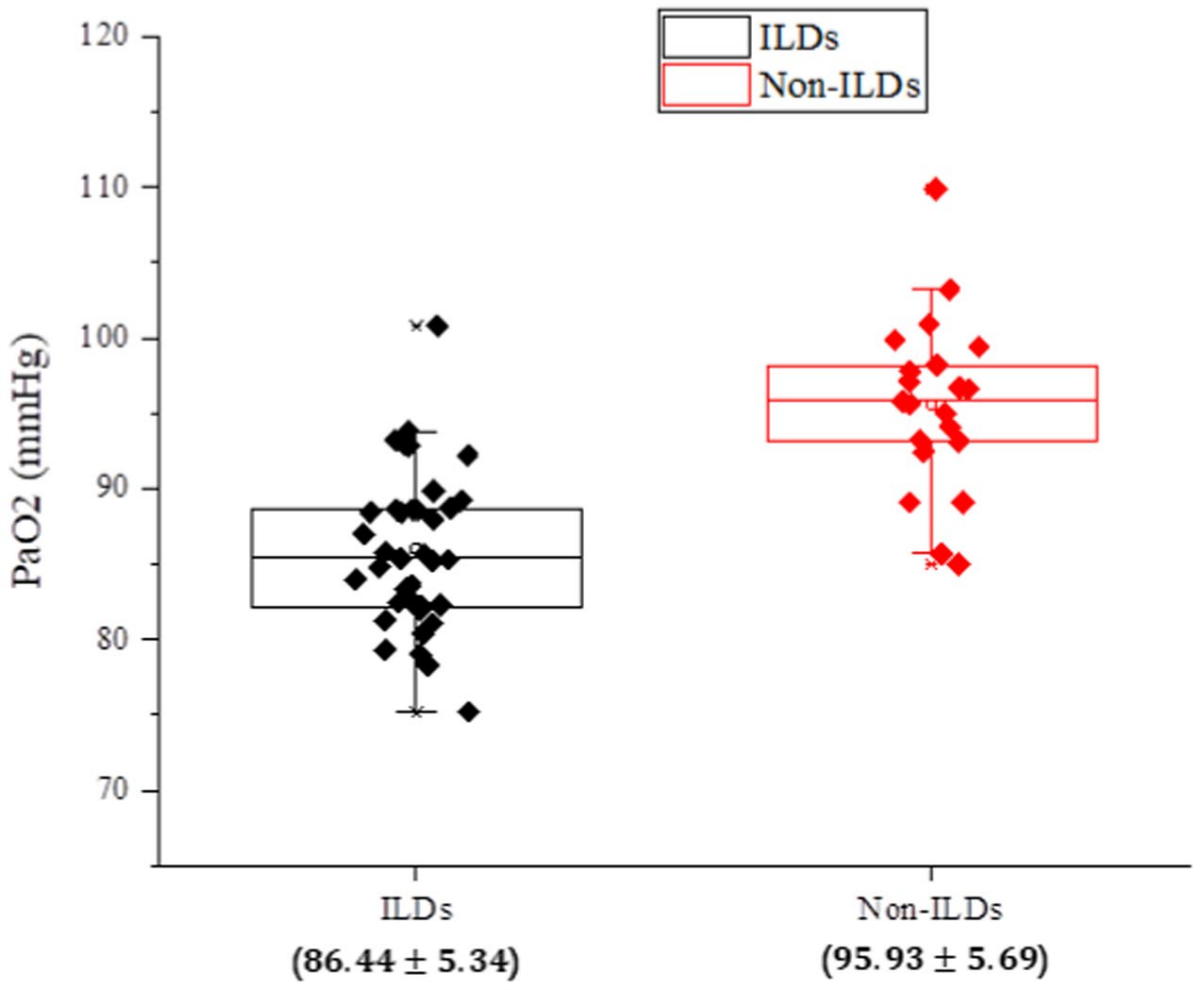
Analysis of PaO2, an index of pulmonary gas functionality, in the experimental and control groups of IPF-ILD.

According to the data illustrated in Figure 1, it can be clearly observed that the vast majority of patients with IPF-ILD show lower values in the index of PaO2 pulmonary gas function, compared to non-ILD subjects, under the same conditions of clinical parameters. This result is a reminder of the significant impact of IPF-ILD on pulmonary gas function. Although PaO2 values of individual normal individuals may be lower than those of IPF-ILD patients, this may be attributed to factors such as inter-individual differences, physiologic characteristics, and testing errors. Overall, however, patients with IPF-ILD did show a significant trend toward decreased lung gas function, which may be related to disease characteristics and structural changes in the lungs. This finding emphasizes the detrimental effects of IPF-ILD on pulmonary air function and provides an important theoretical basis for further research and treatment.

**Figure 2:**
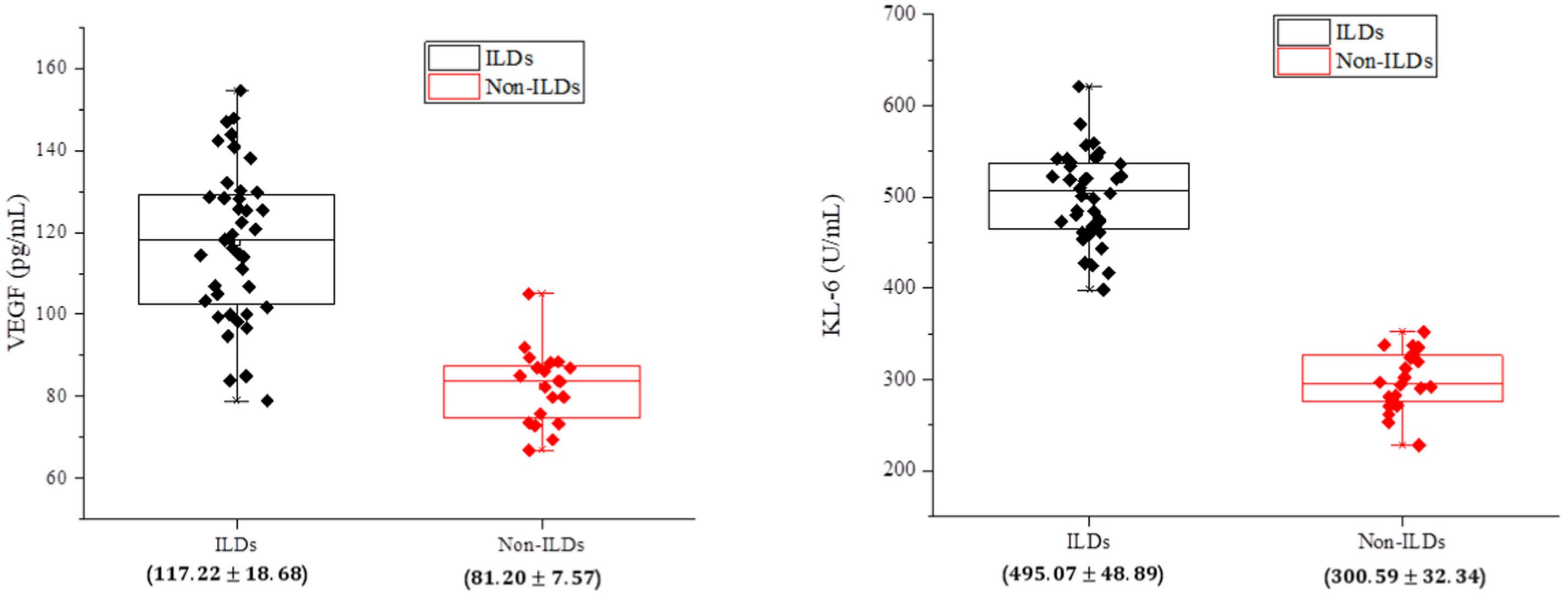
Analysis of serum biological indicators of patients in the experimental and control groups of IPF-ILD.

As shown in Figure 2, it was observed that the expression levels of VEGF and KL-6 were significantly higher in the serum of IPF-ILD patients. This is consistent with previous findings, emphasizing the important roles of VEGF and KL-6 in the pathogenesis of IPF-ILD. Elevated VEGF, as an angiogenic factor, can promote neovascularization and increase vascular permeability, which in turn plays an important role in the pathological process of IPF-ILD. As a protein mainly expressed in type II alveolar epithelial cells and bronchial epithelial cells, the elevation of KL-6 was positively correlated with the degree of tissue fibrotic injury, suggesting that KL-6 may play a key role in the fibrotic process of ILD.

**Figure 3:**
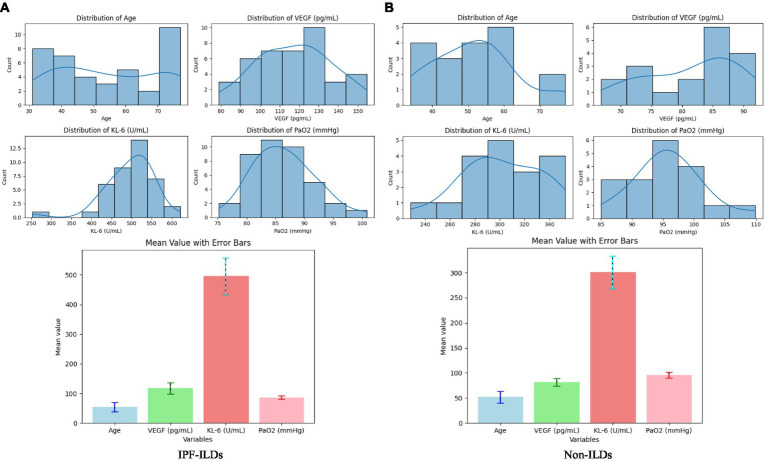
Data distribution of serum biological indicators (VEFG and KL-6), pulmonary gas function indicator (PaO2), and basic clinical parameters (patient age) indicators in patients in the experimental and control groups of IPF-ILD. **(A)** Distribution of clinical parameters for IPF-ILDs, **(B)** Distribution of clinical parameters for Non-ILDs.

**Figure 4:**
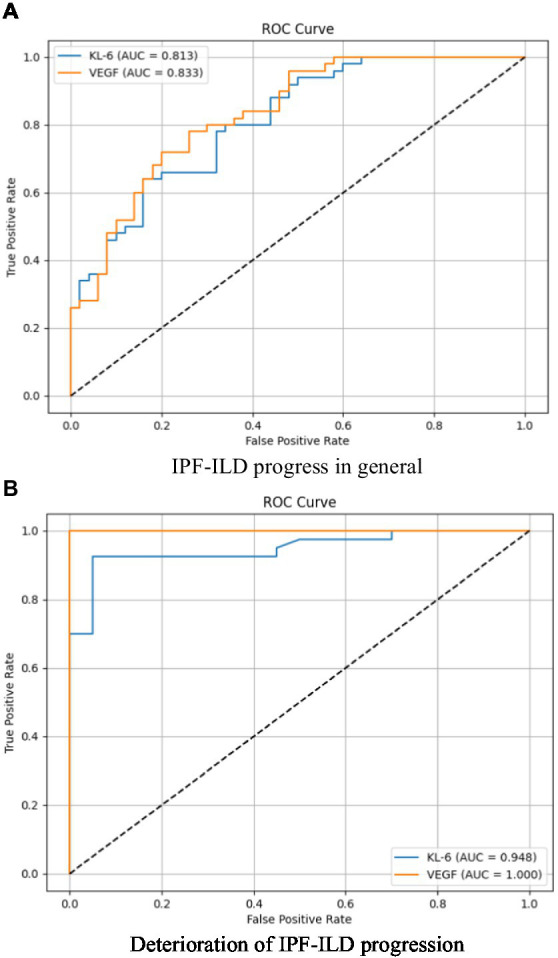
Analysis of the specificity and sensitivity of two important serum bioindicators for pulmonary gas function indicator under different IPF-ILD progression. **(A)** IPF-ILD progress in general, **(B)** Deterioration of IPF-ILD progression.

Based on the data illustrated in Figure 3, it could be observed that the subjects’ measurements were normal or slightly skewed in most cases, except for the age of the patients. The age of the IPF-ILD patients was concentrated around 70 years old, whereas the age of the control group was predominantly below 60 years old. Further analyzing the serum expression levels, it can be observed that the VEGF values of IPF-ILD patients were mainly distributed around 130 *pg/mL*, while those of the control group were mainly distributed around 85 *pg*/*mL*, which indicates that the serum VEGF levels of IPF-ILD patients were relatively high. In addition, the KL-6 values in IPF-ILD patients were mainly distributed around 500 *U*/*mL*, while those in the control group were mainly distributed around 300 *U*/*mL*. This suggests that the serum level of KL-6 is also relatively high in IPF-ILD patients. In terms of pulmonary gas function indexes, the PaO2 values of IPF-ILD patients were mainly distributed around 85 mmHg, while those of the control group were mainly distributed around 95 mmHg. This indicates that under the same clinical conditions, the pulmonary gas function of IPF-ILD patients was significantly impaired, which was manifested by lower PaO2 values. In summary, through in-depth analysis of the data in Figure 4, we clarified the differences between IPF-ILD patients and controls in terms of age, serum VEGF and KL-6 levels, and lung gas function indicators.

### 3.2 Sensitivity and specificity analysis

The purpose of this subsection was to assess the correlation and discriminative power of serum markers KL-6 and VEGF on PaO2, an index of pulmonary air function in subjects with IPF-ILD. The study focused on examining the overall correlation, sensitivity, and specificity between KL-6 and VEGF and PaO2 in order to understand their performance during the progression of IPF-ILD. Through ROC curve analysis, the sensitivity and specificity profiles were specifically compared between two scenarios of IPF-ILD with average and worsening disease degree. As shown in Figure 4, we found that the area under the curve (AUC) of the subject operating characteristic curves (ROC) of the two serum markers when the IPF-ILD disease was in general condition was 0.813 and 0.833, respectively, which indicated that these serum markers had some ability to differentiate between the degree of severity of the IPF-ILD disease in general and were helpful in providing preliminary predictive information. When the IPF-ILD disease was in the worsening stage, the AUC of the ROC curve of the two serum markers were 0.948 and 1.000, respectively. This indicated that these serum markers had higher predictive ability under the worsening stage. Among them, the second serum marker had an AUC value of 1.000, implying that it had perfect accuracy in differentiating between deteriorating and non-deteriorating samples. This finding has important clinical implications as it can provide a reliable guide for early identification of IPF-ILD deterioration, prompting timely therapeutic adjustments and management.

### 3.3 Correlation of VEFG and KL-6 with clinical parameters

**Figure 5:**
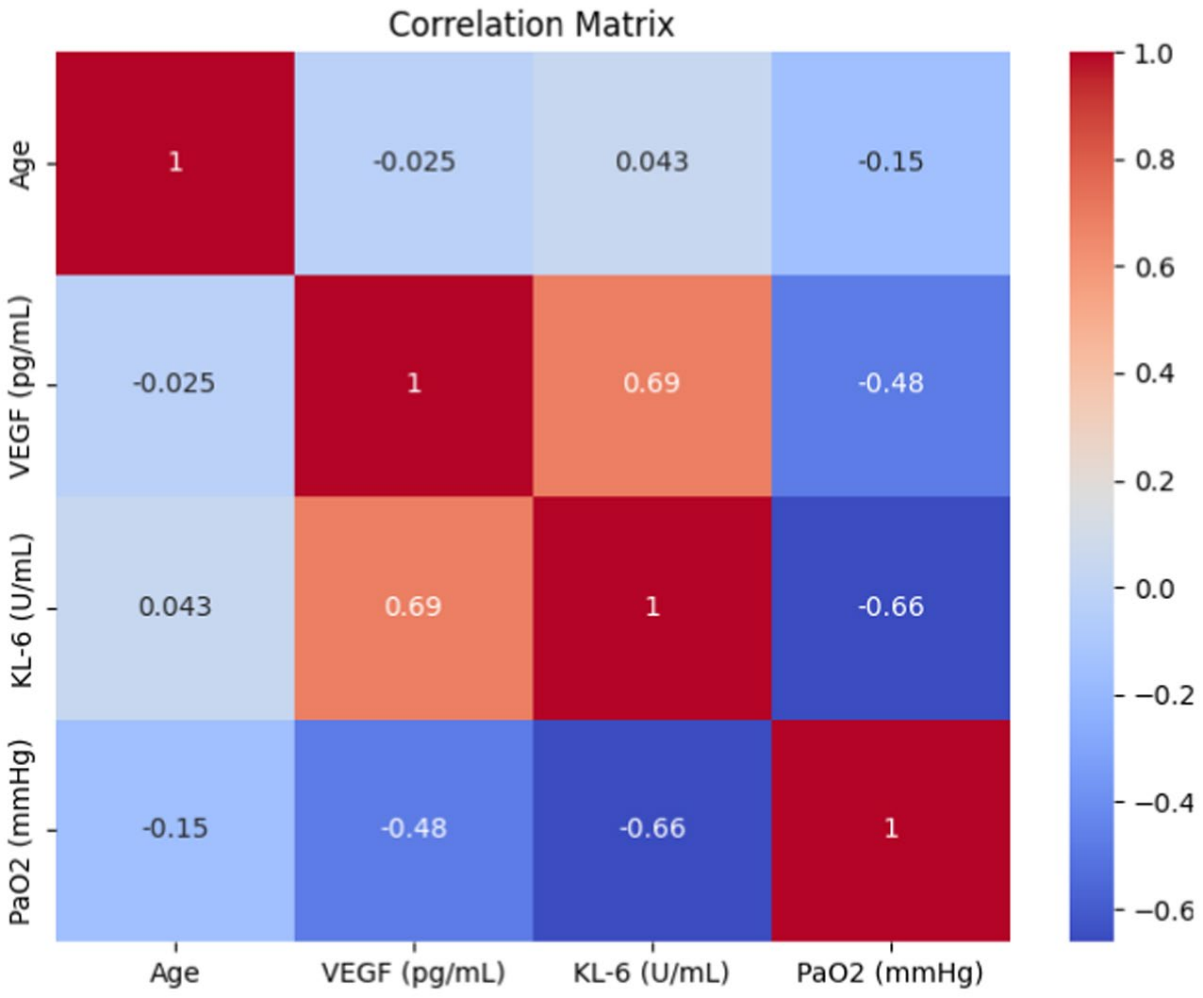
Results of correlation analysis between two serum expression levels (VEFG and KL-6) and clinical parameters.

Subsequently, we performed a correlation analysis of clinical parameters associated with IPF-ILD, focusing on serum VEGF and KL-6 levels, and calculated Pearson’s correlation coefficients between bioindicators and the pulmonary function index PaO2. Through an in-depth interpretation of the analyzed results (Details can be found in Figure 5), first, we observed that serum VEGF levels were negatively correlated with the duration of IPF-ILD (*R* = −0.48, *p* < 0.05). This finding suggests that the course of IPF-ILD may become slower with increasing serum VEGF levels, suggesting a potential role of VEGF in the progression of IPF-ILD. Second, we found a possible negative correlation between KL-6 and PaO2, an index of lung function (*R* = −0.66, *p* < 0.005). This suggests that high levels of KL-6 are strongly associated with lung function impairment in IPF-ILD patients. Specifically, higher KL-6 levels were associated with lower PaO2 values, implying that KL-6 plays an important role in lung damage and functional decline in IPF-ILD.

By analyzing the association of VEGF and KL-6 with related clinical parameters, we found that they were closely related to the severity and prognosis of IPF-ILD. Among the lung function indexes of the patients, the expression levels of VEGF and KL-6 were positively correlated with the decline in lung function, suggesting that both of them may be related to the impairment of lung function in IPF-ILD. In addition, we also found that the expression levels of VEGF and KL-6 were positively correlated with disease duration, and this finding well illustrates that these two biomarkers may be associated with disease progression in IPF-ILD.

**Figure 6:**
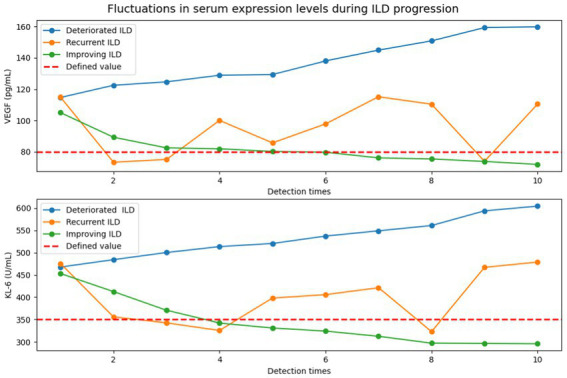
Detection of VEGF and KL-6 serum expression levels in the progression of IPF-ILD (The defined values of serum expression levels of IPF-ILD were first defined by HRCT results, followed by periodic testing of VEGF and KL-6 serum expression level values by chemical assays under three different IPF-ILD progressions (i.e., deteriorated, recurrent, and improving). In this case, the time units on the *x*-axis indicate the different detection time points of the cases, rather than the traditional months or years. This is due to the retrospective nature of data collection in this study, which resulted in inconsistent detection time points between cases and time intervals. This uneven time points reflect the actual data collection to show the longitudinal changes in IPF-ILD during its progression).

Lung structures and lesions can be clearly visualized by high-resolution computed tomography (HRCT) to better identify the extent of IPF-ILD progression. In Figure 6, based on the results of the qualitative analysis of HRCT, a quantitatively defined value for the serum expression level of IPF-ILD was determined. Subsequently, the serum expression levels of VEGF and KL-6 were periodically detected by chemical analysis.

By comprehensively analyzing the expression changes of VEGF and KL-6 in IPF-ILD and their relationship with disease progression and lung function impairment, we conclude that VEGF and KL-6, as potential biomarkers, can play an important role in the early diagnosis and prognostic assessment of IPF-ILD. These findings provide new ideas and methods for the management and treatment of IPF-ILD. By monitoring the expression levels of VEGF and KL-6, physicians can more accurately assess the disease severity of patients and provide early intervention and treatment, thereby reducing the risk of disease progression and improving the quality of life and prognosis of patients.

## 4 Conclusion and discussion

To comprehensively analyze and explore the significance and clinical application value of serum expression levels of VEGF and KL-6 in interstitial lung disease (ILD). By studying these biomarkers, we expect to improve the accuracy of early diagnosis of ILD, assess the severity of the disease, and predict the prognosis of patients. The results of the study showed that the serum expression levels of VEGF and KL-6 were significantly elevated in patients with IPF-ILD and were positively correlated with the severity of ILD, lung function decline, and the duration of the disease, among other relevant clinical parameters.

Analysis of the experimental data indicated that serum VEGF and KL-6 levels are significantly and positively correlated with HRCT scores, suggesting that elevations of both were associated with the severity of ILD lesions. In addition, we found that elevated serum VEGF and KL-6 were closely associated with impairment of lung function, suggesting that these two markers can be associated with ILD disease progression and lung function decline. In addition, serum VEGF and KL-6 concentrations were also negatively correlated with blood gas analysis parameters such as oxygenation index and partial pressure of carbon dioxide, suggesting that both can be closely related to the oxygenation status and respiratory function of ILD patients. Moreover, the analysis demonstrated that as IPF-ILD progressed, the study observed a significant increase in the serum expression levels of VEGF and KL-6 in patients during the exacerbation phase of IPF-ILD. This is due to the fact that during the inflammatory process, cells produce more VEGF and KL-6, two molecules that are released and increased as inflammatory markers in IPF-ILD. At the same time, during the development of IPF-ILD, the fibrotic process gradually intensifies, resulting in damaged and structurally disturbed lung tissue. This structural disorder further stimulates the cells to produce VEGF and KL-6, which triggers more inflammatory responses and fibrotic processes, creating a characteristic expression pattern in the deterioration phase. In the relapse phase of IPF-ILD progression, it was found that patients’ serum expression levels of VEGF and KL-6 remained high beyond the exacerbation phase. This can suggest the instability of the disease, where despite some remission during treatment, the underlying pathologic mechanisms of the disease remain in place and raise the risk of re-exacerbation. Such persistently elevated serum expression levels reflect the persistence of chronic inflammation and fibrosis, as well as structural damage that has not been fully reversed during treatment. However, during the improvement phase of IPF-ILD, studies shown that patients have significantly lower serum expression levels of VEGF and KL-6. This was associated with disease remission and reversal of the fibrotic process. Decreased serum expression levels during the improvement phase reflect the attenuation of the inflammatory process and the reversal of fibrosis. Therapeutic measures help to suppress the inflammatory response, reduce cellular production of VEGF and KL-6, and reverse the fibrotic process, leading to a decrease in serum expression levels.

These findings are important for our in-depth understanding of the pathophysiological process of IPF-ILD.VEGF, as an angiogenic factor, may accelerate the progression of IPF by promoting neoangiogenesis, increasing vascular permeability, and regulating the expression of fibrosis-associated cytokines in the development of ILD.KL-6, as a salivary liquefaction glycan chain antigen, and its high expression may reflect the type II alveolar epithelial cell regeneration and the extent of lung fibrosis injury. Thus, both VEGF and KL-6 are capable of serving as potential biomarkers that can be combined for early diagnosis of IPF-ILD, assessment of disease severity, and prediction of prognosis.

However, it is also important to recognize the limitations of this study. First, the sample size is relatively small, which may affect the stability and generalizability of the results. Further large sample studies are necessary. Second, the present study was a cross-sectional study, and a causal relationship could not be derived. Future longitudinal studies and clinical trials are necessary to validate the clinical value of VEGF and KL-6 as biomarkers in IPF-ILD. In future studies, in addition to VEGF and KL-6, we should further explore other potential biomarkers to obtain a more comprehensive pathophysiological characterization of IPF-ILD. In addition, the combination of genomics and microbiomics research methods can lead to a better understanding of the roles of genetic and environmental factors in the pathogenesis of IPF-ILD. Meanwhile, the concept of precision medicine is also applied to the management and treatment of IPF-ILD, and individualized therapeutic strategies may improve the prognosis and quality of life of patients.

## 5 Summary and inspiration

1. Idiopathic pulmonary fibrosis interstitial lung disease is a complex interstitial lung fibrosis disease with complex pathogenesis and difficult diagnostic and therapeutic processes. This study focuses on the role of serum markers KL-6 and VEGF in patients with IPF-ILD. These markers are strongly associated with disease progression, with VEGF promoting disease progression and KL-6 positively correlating with the degree of fibrotic injury. These studies contribute to unraveling the pathogenesis of IPF-ILD and providing valuable indicators and therapeutic strategies for clinical diagnosis and prognostic assessment.
2. By evaluating the correlation and discriminative capacity of the serum markers KL-6 and VEGF with the lung gas function index PaO2, the study focused on their performance in patients with IPF-ILD. The overall correlation, sensitivity and specificity of KL-6 and VEGF on PaO2 were further explored to understand their role in the progression of IPF-ILD. This facilitates the assessment of the status of pulmonary gas function and disease severity in patients with ILD to provide a more accurate basis for evaluation and treatment.
3. Potential biomarkers associated with IPF-ILD were identified by studying the relationship between KL-6 and VEGF and PaO2. The identified correlations between serum markers and pulmonary gas function are important for understanding the changes in the condition of patients with ILD disease, the effects of treatment, and predicting the prognosis of patients. By delving into the correlations between these markers, we can improve the diagnosis and management of IPF-ILD and provide earlier and more effective interventions for patients to improve their prognosis and quality of life.

## Data availability statement

The datasets presented in this study can be found in online repositories. The names of the repository/repositories and accession number(s) can be found in the article/Supplementary material.

## Ethics statement

The studies involving humans were approved by the Ethics Committee of the First Affiliated Hospital of Gannan Medical University under the ethical approval number [LLSC2023-125]. Written informed consent for the publication of this article has been obtained from Jiangxi University of Science and Technology and The First Affiliated Hospital of Gannan Medical University. The studies were conducted in accordance with the local legislation and institutional requirements. The participants provided their written informed consent to participate in this study.

## Author contributions

BZ: Funding acquisition, Resources, Investigation, Writing – review & editing. SL: Resources, Visualization, Writing – original draft, Writing – review & editing.
