# Corrigendum: Identifying the link between serum VEGF and KL-6 concentrations: a correlation analysis for idiopathic pulmonary fibrosis interstitial lung disease progression

**DOI:** 10.3389/fmed.2024.1424573

**Published:** 2024-07-02

**Authors:** Bin Zhong, Shuixiang Luo

**Affiliations:** Department of Respiratory Medicine, The First Affiliated Hospital of Gannan Medical University, Ganzhou, Jiangxi, China

**Keywords:** IPF-ILD, serum markers, KL-6, VEGF, PaO2

In the published article, there was an error in the affiliation. Instead of “[Department of Respiratory Medicine, The First Affiliated Hospital of Gannan Medical College, Ganzhou City, Jiangxi Province, China],” it should be “[Department of Respiratory Medicine, The First Affiliated Hospital of Gannan Medical University, Ganzhou, Jiangxi, China].”

The authors apologize for this error and state that this does not change the scientific conclusions of the article in any way. The original article has been updated.

